# Development of an activity assay for characterizing deoxyhypusine synthase and its diverse reaction products

**DOI:** 10.1002/2211-5463.13046

**Published:** 2020-12-08

**Authors:** Elisabeth Kaltenegger, Arunraj S. Prakashrao, Serhat S. Çiçek, Dietrich Ober

**Affiliations:** ^1^ Biochemical Ecology and Molecular Evolution Group Botanical Institute and Kiel Botanic Gardens Christian‐Albrechts‐University Kiel Germany; ^2^ Pharmacognosy Group Pharmaceutical Institute Christian‐Albrechts‐University Kiel Germany

**Keywords:** deoxyhypusine synthase, enzyme kinetics, eukaryotic initiation factor 5A, gene duplication, high‐performance liquid chromatography, polyamines

## Abstract

Deoxyhypusine synthase transfers an aminobutyl moiety from spermidine to the eukaryotic translation initiation factor 5A (eIF5A) in the first step of eIF5A activation. This exclusive post‐translational modification is conserved in all eukaryotes. Activated eIF5A has been shown to be essential for cell proliferation and viability. Recent reports have linked the activation of eIF5A to several human diseases. Deoxyhypusine synthase, which is encoded by a single gene copy in most eukaryotes, was duplicated in several plant lineages during evolution, the copies being repeatedly recruited to pyrrolizidine alkaloid biosynthesis. However, the function of many of these duplicates is unknown. Notably, deoxyhypusine synthase is highly promiscuous and can catalyze various reactions, often of unknown biological relevance. To facilitate in‐depth biochemical studies of this enzyme, we report here the development of a simple and robust *in vitro* enzyme assay. It involves precolumn derivatization of the polyamines taking part in the reaction and avoids the need for the previously used radioactively labeled tracers. The derivatized polyamines are quantified after high‐performance liquid chromatography coupled to diode array and fluorescence detectors. By performing kinetic analyses of deoxyhypusine synthase and its paralog from the pyrrolizidine alkaloid‐producing plant *Senecio vernalis*, we demonstrate that the assay unequivocally differentiates the paralogous enzymes. Furthermore, it detects and quantifies, in a single assay, the side reactions that occur in parallel to the main reaction. The presented assay thus provides a detailed biochemical characterization of deoxyhypusine synthase and its paralogs.

## Graphical abstract

An *in vitro* activity assay was developed to characterize the activity of deoxyhypusine synthase, a highly promiscuous enzyme that is essential for cell proliferation and viability in eukaryotes and a promising therapeutic target to treat cancer. Derivatizing the polyamine substrates and products followed by HPLC‐UV/FLD analyses allows the direct quantification of the diverse reaction products of deoxyhypusine synthase in detail.

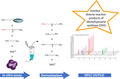

**Abbreviations**
Can: canavalmine
Dah: 1,7‐diaminoheptane
Dap: 1,3‐diaminopropane
DHH: deoxyhypusine hydroxylase
DHS: deoxyhypusine synthase
DTT: dithiothreitol
EDTA: ethylenediaminetetraacetic acid
eIF5A: eukaryotic translation initiation factor 5A
FMOC: 9‐fluorenylmethyl chloroformate
HPLC: high‐performance liquid chromatography
HPLC‐FL: high‐performance liquid chromatography coupled with fluorescence detector
HPLC‐UV: high‐performance liquid chromatography coupled with ultraviolet light detector
Hspd: homospermidine
HSS: homospermidine synthase
i.e.: *id est*
ITSD: internal standard
LC‐MS: liquid chromatography coupled with mass spectrometry
Lys: lysine
NaOH: sodium hydroxide
Put: putrescine
RSD: relative standard deviation
SDS/PAGE: sodium dodecyl sulfate‐polyacrylamide gel electrophoresis
Spd: spermidine
Spm: spermine
*Sv*DHS: DHS from *Senecio vernalis*
*Sv*HSS: HSS from *Senecio vernalis*

Deoxyhypusine synthase (DHS, EC 2.5.1.46) is involved in the post‐translational activation of the eukaryotic initiation factor 5A (eIF5A), a reaction that has two steps [1] (Fig. 1). DHS catalyzes the first step by transferring the 4‐aminobutyl moiety from spermidine (Spd) to a specific lysine residue of the eIF5A precursor protein to form deoxyhypusine [1]. The second step is catalyzed by deoxyhypusine hydroxylase (DHH, EC 1.14.99.29) and involves the hydroxylation of the protein‐bound deoxyhypusine to the unusual amino acid hypusine finally completing the activation of eIF5A [1]. Active eIF5A has been assigned as translation factor with functions in both initiation and elongation [2] and is essential for cell proliferation and viability [3]. In humans, hypusine modification of eIF5A is emerging as a crucial regulator in cancer, infections, and inflammation [4]. Only recently, the active translation factor has been shown to facilitate the sequence‐specific translation of polyproline sequences that otherwise cause ribosome stalling in yeast [5]. In plants, such a sequence‐specific translation is considered to be the basis of the observed involvement of eIF5A hypusination in various physiological processes, including flowering time control, aerial and root architecture, and root hair growth [6]. The genes encoding DHS, DHH, and eIF5A precursor protein are highly conserved in eukaryotes, supporting their fundamental and vital role.

**Fig. 1:**
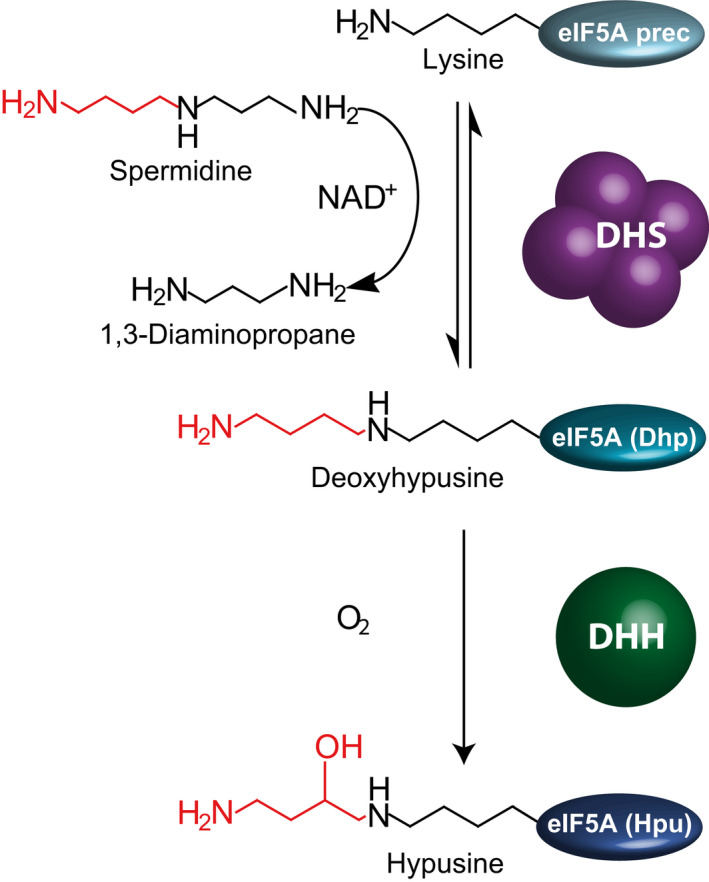
Post‐translational activation of eIF5A. DHS catalyzes the first step of the activation by transferring an aminobutyl moiety from spermidine to a specific lysine residue in the eIF5A precursor protein (eIF5A prec) to form deoxyhypusine (eIF5A(Dhp)). 1,3‐diaminopropane is released. The second step is catalyzed by DHH and involves the hydroxylation of the protein‐bound deoxyhypusine to hypusine (eIF5A(Hpu)).

Deoxyhypusine synthase with its central function in eukaryotic organisms has long been a topic of biochemical research and has also come into prominence for drug development in the treatment of cancer and other diseases [7, 8, 9, 10, 11]. The biologically active unit of DHS is a homotetramer (Figs. 1 and 2), which can be considered as a dimer of dimers [12]. The active sites are formed at the interface of the dimers [12]. In detail, the DHS‐catalyzed reaction uses NAD^+^ catalytically and can be divided into two partial reactions [1]: First, Spd is cleaved by reducing NAD^+^, and the butylamine moiety is transferred to a specific lysine residue (Lys329 in human DHS) [13] of the DHS enzyme to form an enzyme–butylimine intermediate, while 1,3‐diaminopropane (Dap) is released. In the second part, the eIF5A precursor binds to the DHS tetramer. Of note, only a single eIF5A precursor appears to be able to bind to the DHS [12]. The butylimine moiety is then transferred to the ε‐amino group of a specific lysine residue of the eIF5A precursor before it is reduced by recovery of NAD^+^ to deoxyhypusine. Human DHS was shown to be able to catalyze also the reverse reaction. When deoxyhypusine‐containing eIF5A, ^3^H‐labeled in the 4‐aminobutyl portion of its deoxyhypusine residue, was incubated with DHS, NAD^+^, and Dap, [^3^H]spermidine was formed [1].

**Fig. 2:**
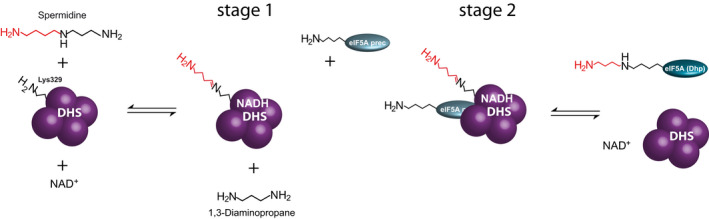
Proposed mechanism of the DHS reaction according to Park *et al*. [34]. The biological active unit of the DHS is a homotetramer, which can be considered as a dimer of dimers. The active sites are formed in the interface of the dimers. The DHS‐catalyzed reaction can be divided into two partial reactions. First, spermidine is cleaved by reducing NAD^+^, and the butylamine moiety is transferred to a specific lysine residue of the DHS enzyme (Lys329) to form an enzyme–butylimine intermediate. 1,3‐diaminopropane is released (stage 1). Secondly, eIF5A precursor binds to the DHS, and the butylimine moiety is transferred to a specific lysine residue in the eIF5A precursor before it is reduced by the recovery of NAD^+^ to deoxyhypusine (stage 2) (eIF5A (Dhp)). It appears that only a single eIF5A precursor (eIF5A prec) binds to the DHS.

Although the main activity of DHS is the activation of eIF5A, DHS from humans and plants are known to be promiscuous with regard to the utilization of aminobutyl donors and acceptors [1, 14, 15]. Specifically, in the absence of the eIF5A precursor, DHS is able to use Spd as both a donor and as an acceptor of the aminobutyl moiety, thereby forming canavalmine (Can) [14] (Fig. 3B). Additionally, human DHS has been described as catalyzing, in the absence of the eIF5A precursor, the NAD^+^‐dependent cleavage of spermidine to generate 1,3‐diaminopropane and a putative 4‐carbon amine intermediate that gives rise to Δ^1^‐pyrroline [13] (Fig. 3B). Furthermore, in the absence of the eIF5A precursor but in the presence of putrescine (Put), DHS can catalyze the transfer of the aminobutyl moiety from Spd to Put yielding homospermidine (Hspd) (Fig. 3A). This side activity has been observed in *in vitro* assays with human DHS and with DHS from various plants [1, 14]. *In vivo*, this side activity seems to be irrelevant in mammals in which the only synthesized polyamines are Put, Spd, and spermine (Spm) [16]. However, in plants, it seems to be present *in vivo*, as Hspd has been detected in various angiosperms [17]. Furthermore, Hspd is an important precursor of pyrrolizidine alkaloids [18], which are secondary metabolites (also called ‘specialized metabolites’) involved in the chemical defense of the plant against herbivores [19, 20]. To date, in all analyzed pyrrolizidine alkaloid‐producing plants, a duplicate of DHS has been recruited to catalyze Hspd as the first step in pyrrolizidine alkaloid biosynthesis [21] (Fig. 3A). These homospermidine synthases (HSS, EC 2.5.1.44) are derived from lineage‐specific independent duplication events in the Asteraceae, Boraginaceae, Convolvulaceae, Fabaceae, and monocots [22, 23, 24]. The macromolecular assembly of DHS into a homotetramer [12] might have been an important aspect in the repeated parallel evolution of HSS. After the duplication of genes that encode homomeric proteins, the duplicates are predicted to interfere at the protein level by forming paralogous heteromers [25]. The way in which this interference shapes the fate of duplicates is the subject of current research [26, 27, 28, 29]. Indeed, it has been shown that in *Trypanosoma brucei* DHS and its paralog can form a tetramer with a highly increased enzyme activity [30, 31]. A similar heterotetrameric architecture is suggested for paralogous DHS proteins from *Leishmania major* [11].

**Fig. 3:**
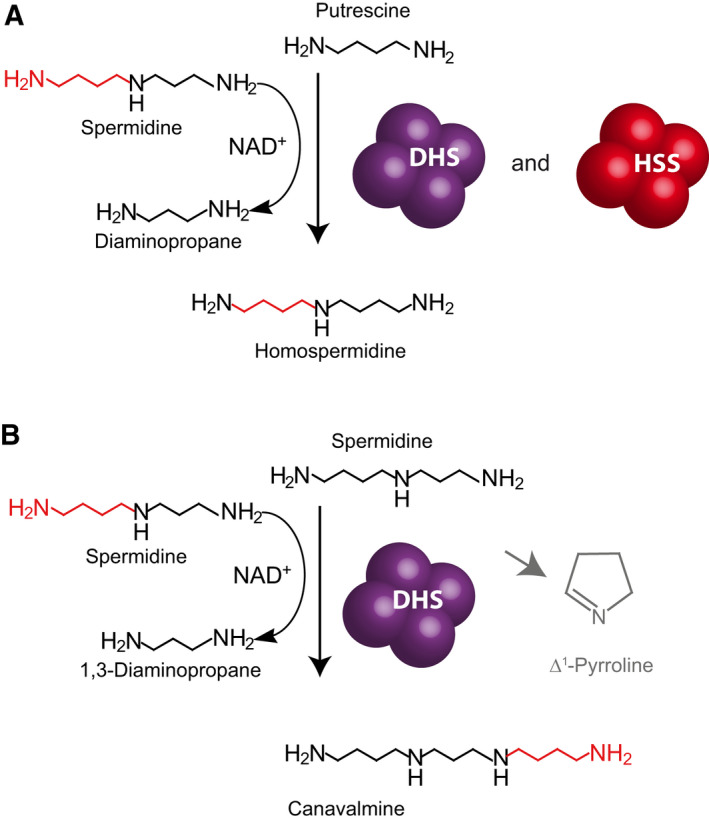
eIF5A analogs as acceptors of the aminobutyl moiety. (A) Homospermidine synthesis. If the aminobutyl moiety of spermidine is transferred to putrescine, the triamine homospermidine is produced. Several DHS paralogs in plants have optimized this function while losing the ancient function and thus evolved into homospermidine synthase (HSS). (B) Spermidine functions as an aminobutyl donor and aminobutyl acceptor. In the absence of eIF5a, DHS can use spermidine as an acceptor and donor of the aminobutyl moiety, thereby producing the tetraamine canavalmine. Furthermore, human DHS has been described to catalyze, in the absence of eIF5a, the NAD‐dependent cleavage of spermidine to generate 1,3‐diaminopropane and a putative 4‐carbon amine intermediate that gives rise to Δ^1^‐pyrroline.

Given the relevance of DHS for essential physiological processes in human, animals, and plants, and given the finding that HSS as a duplicate of DHS is relevant in the biosynthesis of plant secondary metabolism, assays are needed that allow us to characterize and distinguish these enzymes. Several *in vitro* assays have previously been described for the quantification of the biochemical activity of DHS and its paralogs. In the DHS assay, the enzyme’s ability to use the eIF5A precursor as an aminobutyl acceptor is tested (DHS activity), whereas in the HSS assay (an assay for the paralog of DHS), the use of putrescine as an aminobutyl acceptor is characterized (HSS activity) [14, 21, 32, 33]. Many of these assays measure the incorporation of radioactivity from ^3^H‐ or ^14^C‐labeled Spd into the eIF5A precursor protein or other aminobutyl acceptors [32, 33]. However, a huge drawback of these assays is the accumulation of radioactive waste. Additionally, as Park *et al*. [34] have previously highlighted, these assays involve multiple manual steps and are therefore prone to experimental errors. Park *et al*. [34] have designed a new assay, adaptable to high throughput, utilizing the NADH generated in the first partial reaction of the DHS. However, a drawback with this assay is the indirect measurement of activity via the cofactor. Whereas, for routine screening, this assay offers a fast and feasible method to test for enzyme activity, a detailed characterization of DHS and its paralogs with respect to the use of various donors and acceptors for the aminobutyl moiety requires a more sophisticated assay. Here, we present a HPLC‐based approach including 9‐fluorenylmethyl chloroformate (FMOC) precolumn derivatization to monitor the polyamine turnover in *in vitro* assays. FMOC is a common derivatization reagent employed to derivatize amino groups [35]. We have re‐analyzed previously characterized DHS and HSS of *Senecio vernalis* to compare the values with former data [14, 21]. Although focusing on the characterization of the enzyme’s ability to transfer the aminobutyl moiety of Spd to the eIF5A precursor (DHS assay) and Put (HSS assay) as aminobutyl acceptors, we have been able to detect even more promiscuity in substrate usage in the *in vitro* assays than those described in the literature.

## Results

The idea of the new assay was to detect directly and quantify the polyamine reaction products (Figs 1 and 3), without using radioactivity for tracers or coupled assay strategies. For this, the enzyme was incubated with the substrates Spd and eIF5A precursor for the so‐called ‘DHS assay’ or with Put and Spd for the so‐called ‘HSS assay’. After incubation, polyamines in the reaction mixture resulting from the remaining substrate, from product formation, and from the added internal standards were derivatized with FMOC and analyzed by HPLC‐UV/FL. An important step was to establish reproducible FMOC derivatization under the given assay conditions.

### Development of the method—factors affecting the derivatization procedure

Crucial for the derivatization is the solvent in which the analytes are dissolved. Previously, DHS activity assays were carried out in glycine buffer [21, 32]. However, the amino acid glycine is a substrate for FMOC derivatization itself and competes with the polyamines in the derivatization reaction. To avoid this, a borate buffer was tested as an alternative. We intended to establish a derivatization protocol for the relevant di‐ and polyamines Dap, Put, Spd, and Hspd consumed or produced by the enzyme and for Dah and Cad, which were used as internal standards each at concentrations of 40 and 400 µm for the DHS and HSS assays, respectively, in both assay buffers. Not only the reproducibility of absolute and relative peak area but also the ratio of peak size diamine to triamine (2 : 3) was considered important as the intensity of UV absorbance and fluorescence emission correlate with the number of FMOC moieties in the derivatized molecule and thus the amino groups in the polyamines.

The most important factors for successful and reproducible derivatization proved to be the concentration of FMOC, the molarity and pH of the derivatization buffer, the binding of excess FMOC before HPLC separation, and the composition of the solution used to stop the derivatization reaction.

1. *FMOC concentration*: An excess of FMOC needs to be provided in order to guarantee a complete derivatization of the polyamines. A molar ratio of the reactants [FMOC]:[substrate] of at least 3 : 1 proved to be optimal. Of note, when derivatizing samples based on the glycine buffer, the amount of glycine provided by the buffer has to be included in the calculation of the amount of substrate for derivatization. Thus, higher FMOC amounts have to be used in comparisons with samples based on borate buffer.
2. *Molarity and pH of the derivatization buffer*: Borate buffer is commonly used for FMOC derivatization [35]. Optimal derivatization results documented by a good response for all polyamine FMOC signals were obtained in 0.6 m borate buffer at pH 8. If the pH of the derivatization buffer was lower than pH 7, no derivatization occurred at all. Moreover, if the molarity of the derivatization buffer was too low, polyamines were not homogenously derivatized, meaning that the ratio between the polyamines in the derivatization replicates was not stable.
3. Binding of excess FMOC by adding piperidine: Free FMOC molecules resulted in artifacts if they were not removed from the derivatization mixture before HPLC separation. These artefacts were possibly attributable to the spontaneous hydrolysis of the FMOC adducts, as described by Ref. [36]. Thus, excess piperidine was added after the derivatization of the polyamines to react with the unused FMOC. Piperidine elutes first from the HPLC column and did not interfere with the signals of the FMOC–polyamine derivatives.
4. *Composition of the solution to stop the derivatization reaction*: The ‘stop solution’ was optimized by including tetrahydrofuran or dimethylformamide to improve the solubility of the hydrophobic FMOC–polyamine derivatives. Dimethylformamide is favored over tetrahydrofuran in order to improve reproducibility in the detection of the derivatized samples, as tetrahydrofuran might eventually evaporate because of its volatile nature. Furthermore, sodium acetate buffer (400 mm, pH 5) was added to the stop solution to shift the pH of the sample to 7 prior to injection for enhanced column lifetime [37].

### Chromatographic separation and reproducibility

**Fig. 4:**
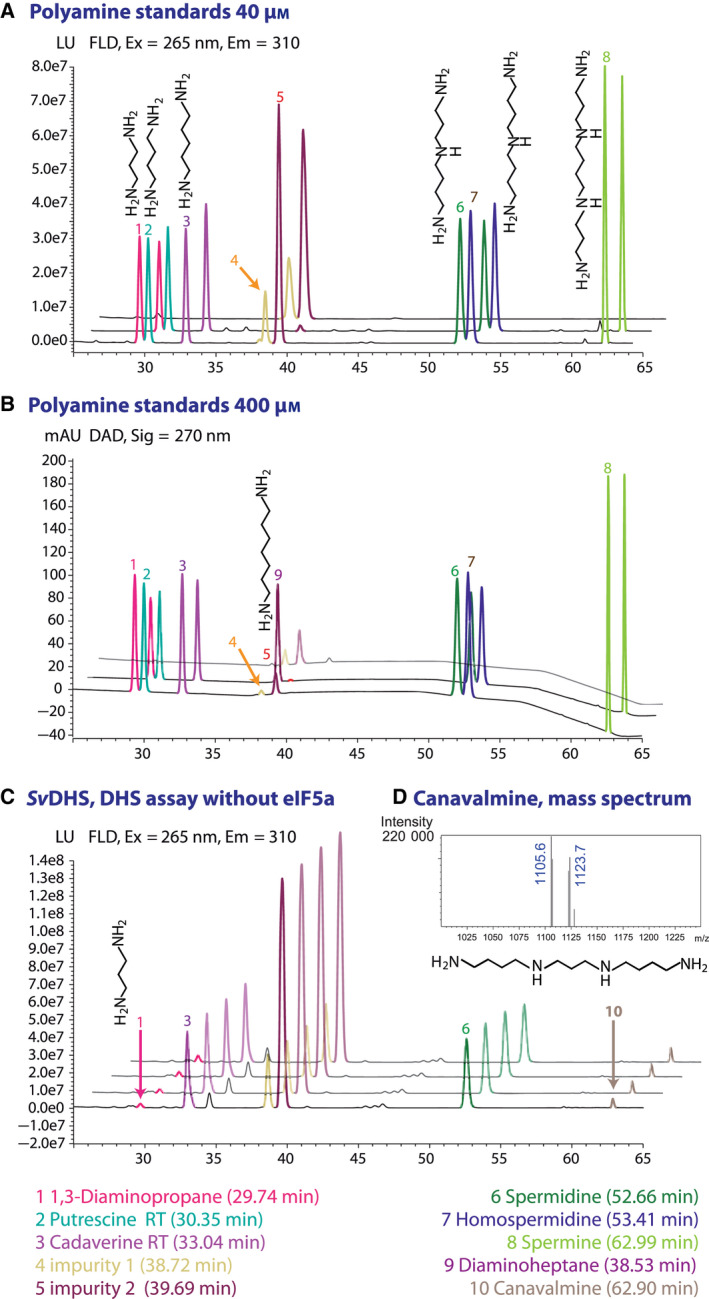
HPLC chromatograms of polyamines. Polyamine structures are given without FMOC moiety. (A) Mixtures of polyamine standards (Dap, Put, Spd, Hspd, Spm, 40 µm) were derivatized with FMOC as optimized for DHS assay samples with Cad added as an internal standard. Front trace, polyamine standard in glycine buffer; middle trace, polyamine standard in borate buffer; rear trace, a derivatization blank of glycine buffer plus precipitation solution without internal standard. (B) Analog to A, but polyamine standards in a concentration of 400 µm, each, were derivatized as optimized for HSS assay samples with Cad as an internal standard. Again, no internal standard was added in the derivatization blank of the glycine buffer (rear trace). The polyamine standard mixture in borate buffer also included Dah as an internal standard (middle trace). (C) DHS assay without eIF5a of *Sv*DHS. From the front to the back trace, samples after 1, 2, 4, and 8 min of incubation time are shown. Dap and Can increase slightly over time. (D) Zoom of the mass spectrum of canavalmine. *m*/*z* ratios of 1105 and 1123 correspond to the [M + H]^+^ and [M + H_2_O + H]^+^ ions of FMOC Can, when all four amino groups are derivatized. The full mass spectrum is given in Fig. S3.

The separation of the FMOC derivatives was optimized using a mixture of polyamine standards (Dap, Put, Spd, Hpsd, Spm) in concentrations of 40 and 400 µm. Spm was also included as commercially available tetraamine and to substitute for Can. Figure 4 shows typical chromatograms of mixtures of these standards in glycine‐ and borate‐based assay buffers analyzed after derivatization according to the optimized protocol. Chromatographic conditions were optimized to give a good separation of the structurally highly related pairs Dap/Put and Spd/Hspd, which differ only by one C‐atom in the carbon chain. Derivatization blank measurements, meaning that only assay buffer mixed with acetonitrile was derivatized with FMOC in acetone, revealed several impurities. Minor impurities co‐elute with Put and Spd and thus affect the detection limit of polyamines under UV and FL detection (Table 1). For example, although a minimum amount of Dap, Hspd, and Spm at 0.03 pmol is detected with FL, the detection limit of Put and Spd is increased to 2 pmol (Table 1). The most prominent impurities, however, eluate with Dah (impurity 1) and close to Dah (impurity 2) (Fig. 4). The amount of these impurities correlates with the amount of FMOC dissolved in acetone, which is added for the derivatization reaction. For samples deriving from the DHS and HSS assays in the borate buffer, for which only small amounts of FMOC (36 and 180 nmol, respectively; Table 3) were used in the derivatization reaction, impurity 1 is negligible being <0.5% of the internal standard Dah. However, to derivatize samples deriving from assays in glycine buffer, the amount of FMOC is increased to 1800 nmol to compensate for the excess of glycine. For samples derived from HSS assays, in which the polyamines and the internal standard have concentrations of 400 µm each, impurities increase to approx. 7%. For samples derived from DHS assays with polyamine concentrations of only 40 µm each, impurities even increased to approx. 28% of the internal standard Dah, because of the unfortunate combination of high FMOC concentrations and low concentrations of polyamines. Thus, the borate‐based assay buffer should be favored to reduce the FMOC‐derived impurities. If the glycine‐based assay buffer is used, Cad should be used as an internal standard because it does not co‐elute with any of these impurities (Fig. 4).

**Table 1:** Linearity of polyamine detection under assay conditions with HPLC‐UV and HPLC‐FL. Linearity of relative peak size and detection limit are summarized from a seven‐level calibration. Two different assay buffers were analyzed, a borate‐based assay buffer (B) and a glycine‐based assay buffer (G).

Polyamine	Detection limit [pmol]		Detection range [pmol]		Slope of calibration graph		Correlation coefficient	
	B	G	B	G	B	G	B	G
Dap								
UV	2	2	2–483	2–465	0.218	0.221	0.989	0.999
FLD	0.03	0.025	0.03–20	0.25–20	4.104	5.268^a^	0.9955	0.9982
Putrescine								
UV	2	2	2–483	2–465	0.198	0.200	0.9926	0.9992
FLD	2	0.5	2–20	0.5–20	4.475^b^	5.245^c^	0.9999	0.9989
Spermidine								
UV	9	4	9–483	4–465	0.283^b^	0.287^a^	0.9916	0.9998
FLD	2	0.03	2–20	0.03–20	6.245^b^	6.513	0.9994	0.996
Homospermidine								
UV	2	2	2–483	2–465	0.266	0.293	0.9942	0.9991
FLD	0.03	0.03	0.03–20	0.03–20	6.766	7.353	0.9995	0.9971
Spermine								
UV	2	2	2–390	2–200	0.418^c^	0.414^b^	1.000	0.9979
FLD	0.14	0.14	0.06–20	0.06–20	9.257	9.738	0.9988	0.9981

^a^ 6‐level calibration.

^b^ 4‐level calibration.

^c^ 5‐level calibration.

The reproducibility of polyamine detection by HPLC‐UV/FL after derivatization was examined by first comparing three independent injections from one sample. The absolute peak areas of the derivatized polyamines showed a relative standard deviation (RSD) below 2% in borate buffer and in glycine buffer. When comparing peak area relative to the ITSD, the RSD dropped below 1%. Second, independent derivatization replicates of polyamine standards in borate buffer (Dap:Put:Spd:Hspd, 40 and 400 µm, each) and glycine buffer (Put:Spd, 40 and 400 µm, each) were analyzed and showed an RSD of the relative peak area from 3.0 to 11.5%, reflecting the error introduced by pipetting and derivatization (see Fig. S1). Detection linearity relative to the ITSD is given in both, the borate buffer and the glycine buffer, in the investigated concentration range for all polyamines, except Spm (slope calibration graph, Table 1). The linearity range for Spm was slightly narrower, and saturation was reached at 200 pmol in glycine buffer. Furthermore, the ratio for the slopes of the calibration graphs reflected the number of amino groups per polyamine, indicating the full derivatization of the polyamines and supporting the assumption that the intensity of UV absorbance and fluorescence emission correlate with the number of FMOC moieties in the derivatized molecule.

### Possible co‐precipitation of polyamines

To stop the enzyme reaction and to quantify the polyamines present in the assay reactions, it was necessary to remove the proteins (enzyme and, in the case of the DHS assay, the remaining eIF5A precursor) by precipitation before derivatization. However, polyamines are, because of their ionic nature, predicted to interact nonspecifically with proteins [38] and thus might be co‐precipitated in this step. This might be particularly relevant under the conditions of the assay at pH 9, as polyamines are protonated and have a cationic nature, whereas *Sv*DHS and *Sv*HSS with isoelectric points of 5.0 and 5.3 are predicted to be negatively charged. To test for the possible co‐precipitation of polyamines with the protein, a polyamine standard mixture in 50 mm borate buffer at pH 9 was treated in two ways: First, the polyamine mixture was directly mixed with the precipitation solution (acetonitrile containing the internal standard), and second, the polyamine mixture was mixed with 40 µg *Sv*HSS and incubated for 2 min before the precipitation solution was added. By omitting the catalytically important cofactor NAD^+^, a conversion of polyamines because of the reduced activity of HSS could be neglected. The polyamine standard mixture contained Spd (40 µm) plus Dap and Spm as relevant possible products in the DHS assay at lower the concentrations (8 µm, each). Spm was used to substitute for Can, which is not commercially available. Both treatments were derivatized and analyzed by HPLC‐FL. When comparing the relative peak areas of the derivatized polyamines in the two samples, the observed difference was 1–7%, that is, in the range of the error of independent derivatization replicates. Thus, the co‐precipitation of polyamines with the protein, which might affect their quantification, was excluded under the optimized precipitation conditions.

### DHS activity assays with recombinant DHS and HSS enzymes

For comparison, the DHS activity, that is, the ability of the previously characterized DHS and HSS from *S. vernalis* (*Sv*DHS, *Sv*HSS) to use the eIF5A precursor as an aminobutyl acceptor [14], was tested with the new assay in the glycine‐based and in the borate‐based assay buffers. The enzymes were heterologously expressed and affinity‐purified (Fig. S2). The specific enzyme activities were calculated based on the increase in the product Dap, which was quantified via its fluorescence emission relative to the internal standard (Table 2). Of note, if the eIF5a precursor protein is modified by the enzyme, equimolar amounts of Dap are produced (Fig. 1).

**Table 2:** Specific activities of *Sv*DHS and *Sv*HSS enzymes in borate‐ and glycine‐based buffer were calculated from product formation quantified by HPLC‐UV and FLD detection. Three technical assay replicates were performed, and the relative standard deviation is given in brackets. B indicates borate‐based assay buffer, while G indicates glycine‐based assay buffer.

Enzyme		DHS assay Aminobutyl acceptor eIF5a (pkat·mg^−1^)	HSS assay Aminobutyl acceptor putrescine (pkat·mg^−1^)		
Product		Dap	Dap	Hspd	Can
DHS *S. vernalis*	B	338 (15%)	203 (14%)	133 (4%)	60 (9%)
	G	508 (12%)	545 (11%)	341 (8%)	175 (21%)
HSS *S. vernalis*	B	n.a.	856 (9%)	848 (14 %)	n.a.
	G	n.a.	317 (19%)	289 (16%)	n.a.

[Correction added on 24 December 2020, after first online publication: table 2 corrected because only the first 'Dap' column corresponds to the DHS assay]

**Fig. 5:**
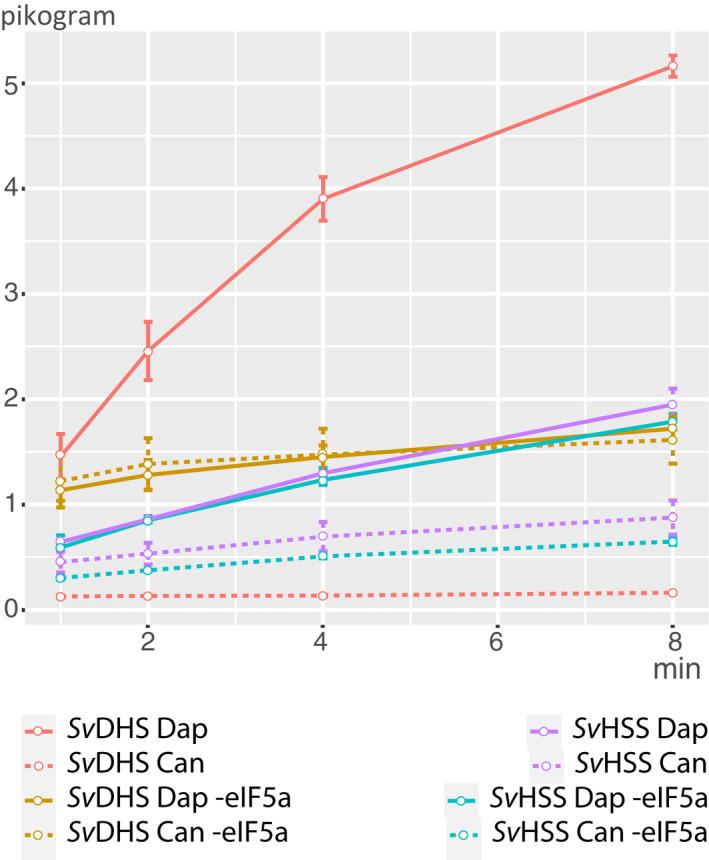
DHS assay. Product increase (Dap and Can) is shown over time for *Sv*DHS and *Sv*HSS. The standard DHS assay includes spermidine as aminobutyl donor and the eIF5a precursor as aminobutyl acceptor. Additionally, a second assay was performed in which eIF5a was omitted, indicated as ‘‐eIF5a’ in the legend. Standard error of three assay replicates is shown.

For *Sv*DHS, Dap initially increased linearly (Fig. 5), and the specific activities of *Sv*DHS in the borate‐based and the glycine‐based assay buffers were calculated to be 338 and 508 pkat·mg^−1^, respectively (Table 2). This is an approx. tenfold higher activity than the previously described 58 pkat·mg^−1^ [14]. The RSD of the specific activities was 15 and 12% of three replicates in the two buffer systems, respectively (Table 2). Thus, the new DHS assay delivers robust and reproducible data to determine DHS activity and seems to be even more sensitive than the previously used assay.

*Sv*HSS, which was described as not being active in the DHS assay [14], showed, under the same assay conditions, a much slower Dap increase than *Sv*DHS, and concomitantly, a second peak with the retention time close to that of Spm increased in size (Fig. 5). This pattern was observed in both the borate‐based and the glycine‐based assay buffers (each in triplicate). *Sv*DHS and *Sv*HSS are known to utilize Spd not only as an aminobutyl donor, but also as an aminobutyl acceptor, thereby forming Can [14] (Fig. 3B). Because no commercial reference standard was available for Can (see also above), we used LC‐MS to verify the nature of this compound. Indeed, *m*/*z* ratios of 1105 [M + H]^+^ in the positive ionization mode and 1149 [M + HCOO]^−^ in the negative mode were detected (Fig. S3), data that are consistent with the expected mass of a fully derivatized molecule of Can. Thus, *Sv*HSS utilizes Spd as an aminobutyl acceptor under the conditions of the DHS assay. Based on the reaction mechanism (Fig. 3B), Dap and Can should be produced in equimolar amounts if the synthesis of Can is the only reaction product occurring under these assay conditions. However, the amount of produced Dap was clearly higher than Can (Fig. 5, ratio 1 : 0.4), indicating either that *Sv*HSS also transfers the aminobutyl moiety to the eIF5A precursor protein, although at a very low rate, or that the Spd molecule is solely cleaved, thereby producing Dap and Δ^1^‐pyrroline (Fig. 3B). Therefore, the activity of *Sv*HSS was analyzed again under identical assay conditions, but without the eIF5A precursor. *Sv*HSS produced Dap and Can in a similar pattern as that detected in the presence of the eIF5A precursor (Fig. 5). Although only a small amount of product was observed, the amount of Dap was constantly higher than that of Can (ratio 1 : 0.4 to 1 : 0.5), suggesting that Spd is indeed cleaved under these assay conditions. Notably, *Sv*DHS also produced Dap and Can at a slow rate in the absence of eIF5A precursor (Fig. 5), but here the ratio of Dap:Can ranged from 1 : 0.8 to 1 : 0.9, which indicates that the transfer of an aminobutyl moiety from one Spd molecule to another Spd molecule is the main reaction.

Summarizing, the new assay allowed us to determine DHS activity robustly via Dap quantification. Additionally, the side reactions that occurred in the DHS assay, such as the use of Spd as an aminobutyl donor and an acceptor and the sole Spd cleavage, could be quantified by comparing the enzyme activity in the presence and absence of the eIF5a precursor protein and by comparing the amount of the produced polyamines.

### Detection of deoxyhypusine

**Fig. 6:**
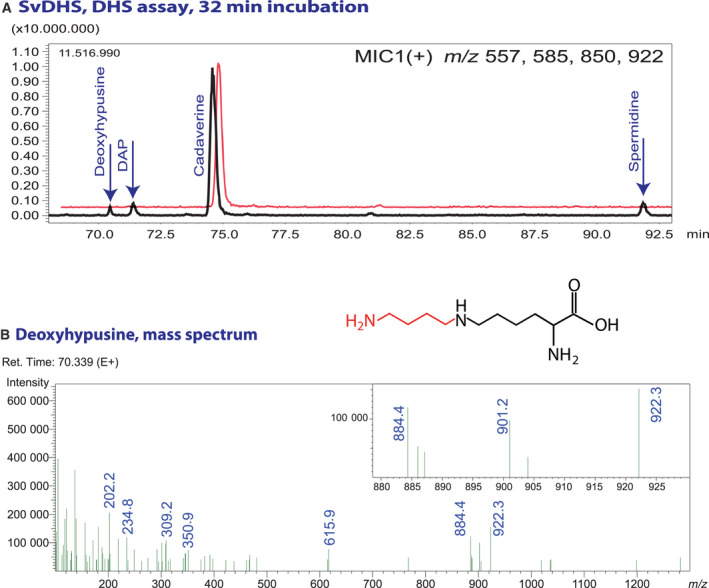
Detection of deoxyhypusine. (A) Multiple ion chromatogram of an DHS assay of SvDHS with and without spermidine. The reaction mixture was hydrolyzed and derivatized with FMOC. The range of the chromatogram including deoxyhypusine, Dap, Cad, and Spd is shown. (B) Mass spectrum of deoxyhypusine. *m*/*z* ratios of 884, 901, and 922 correspond to the [M + H]^+^, [M + NH_4_]^+^, and [M + K]^+^ ion of FMOC deoxyhypusine, when all three amino groups are derivatized.

As a proof of concept, the DHS assay protocol was modified to detect deoxyhypusine. It includes acid hydrolysis of the proteins after the typical *in vitro* enzyme reaction followed by derivatization of the amino acids and polyamines in the reaction mixture. LC‐MS was used to identify deoxyhypusine. Figure 6 shows the multiple ion chromatogram (MIC) of an assay after 32 min of incubation of *Sv*DHS with eIF5a precursor protein and Spd in comparison with an assay without Spd as negative control. *m*/*z* ratios of 557, 585, 850, and 922 representing [M + K]^+^ ions of derivatized Dap, Cad, Spd, and deoxyhypusine were monitored. The presence of deoxyhypusine was confirmed by *m*/*z* ratios of 884, 901, and 922 corresponding to [M + H]^+^, [M + NH_4_]^+^, and [M + K]^+^ ions of the fully derivatized compound. In the assay reaction, both Dap and deoxyhypusine could be detected as product along with Spd as substrate.

### HSS activity assays with recombinant DHS and HSS enzymes

*Sv*HSS and *Sv*DHS were additionally characterized in the HSS assay by using Put as an acceptor for the aminobutyl moiety from Spd in both buffer systems. Because of the higher amounts of Spd and Put as substrates (400 µm, each) in this assay, the amounts of produced Dap and Hspd allowed quantification by UV absorption (Fig. 3A). A typical initial linear phase of product formation could be observed in the assays with *Sv*HSS. The amount of produced Dap correlated closely with Hspd, as reflected in the highly similar specific activities calculated for both products, which were 856 and 848 pkat·mg^−1^, respectively (Table 2). This indicated that no side activity had an impact on the analyses (Table 2). In the glycine‐based buffer assays, the specific activities were generally lower than those in the borate buffer (Table 2). The calculated specific activities of three replicates showed 9–19% RSD, confirming that the new HSS assay provides robust and reproducible data similar to the previously reported specific activity of 784 pkat·mg^−1^ [14]. For *SvDHS*, the calculated specific activities were higher in glycine‐based buffer assay than in the borate‐based buffer assays, but the most important difference between the two enzymes was that *Sv*DHS produced not only Hspd, but also Can in the HSS assay. In other words, *Sv*DHS used both Put and Spd as aminobutyl acceptors under these assay conditions, although it favored Put, as reflected by the specific activity calculated from Hspd formation, which was 138 pkat·mg^−1^ versus 60 pkat·mg^−1^ when calculated from Can. Thus, the new HSS assay allowed us to detect and quantify all polyamines produced in the *in vitro* assay and also revealed when Spd was utilized as an aminobutyl donor and an acceptor by the enzymes.

### Substrate competition studies of *Sv*DHS

*Sv*DHS shows flexibility with regard to the utilization of aminobutyl acceptors, as it can use the eIF5A precursor, Put, and Spd. To test which acceptor is the most favored one, competition studies of *Sv*DHS were performed in a third assay type in which the eIF5A precursor, Spd, and Put (each 40 µm) were incubated with *Sv*DHS. Previously, a competitive inhibition of the aminobutylation of the eIF5A precursor by Put was reported when Put was provided in excess [14]. In the present competition assay, Dap increased linearly, and with 240 (RSD 25%) and 564 pkat·mg^−1^ (RSD 9%) in the borate and in the glycine buffers, respectively, the specific activities were calculated to be in the range of the ‘standard’ DHS assay described above (Table 2). Of note, neither Hspd nor Can was produced. Thus, *Sv*DHS preferentially catalyzes the aminobutylation of the eIF5A precursor in the presence of equal amounts of aminobutyl acceptors. Only if the eIF5A precursor was absent other acceptors were utilized by *Sv*DHS.

## Discussion

In the current study, a new assay was developed to characterize DHS and its paralogs by quantifying directly the amount of the polyamine products without the need of radioactively labeled tracers. The polyamines are detected via UV absorption or FL detection after derivatization with FMOC. FMOC is a commonly used derivatization reagent for amino acid [35] and polyamine [36, 39] analyses and reacts with primary and secondary amines. Compared to dansyl chloride and benzoyl chloride, which are also commonly used reagents for detection of polyamines, FMOC is the at least toxic derivatization agent and provides a fast derivatization reaction in aqueous solutions at ambient temperature [35]. The fluorescence spectrophotometry of FMOC derivatives provides a detection limit of 0.03–2 pmol (Table 1), which is comparable to dansyl polyamines [40]. *O*‐phthalaldehyde (OPA), that specifically reacts with primary amino groups, provides a method that is comparable in its sensitivity to detect polyamines, but OPA derivatives have a limited stability [41].

The intensity of the UV absorption and FL emission in the derivatized molecule is assumed to depend on the amount of FMOC moieties [36]. However, the chemical structure of the fluorophore environment could also affect the intensity of UV absorption and FL emission. While it could be shown that the ratio for the slopes of the calibration graphs follows the ratio of the amino groups in the Dap, Put, Spd, and Spm (Table 1) [36], such a proof is lacking for Can as it is not commercially available.

Quantifying the polyamine products provides a simple assay with a few manual steps and therefore reduces experimental errors. However, while this approach is straight forward when characterizing the activities of the DHS‐like enzymes with its diverse polyamine substrates (Fig. 3), the enzyme genuine substrate—the eIF5A precursor protein—cannot be detected and quantified directly. As the modification of the eIF5A precursor requires Spd cleavage, which is part of the first stage of the complex DHS reaction (Fig. 2) and provides Dap as a byproduct (Fig. 1), Dap can be used to indirectly calculate the enzyme activity with the eIF5A precursor. This cleavage is also utilized in the new nonradioactive assay developed by Park *et al*. [34]. However, one has to consider that Dap can be formed also when Spd is used as aminobutyl donor and acceptor. In this case, Can will be detected by the developed assay. Furthermore, Spd can be cleaved only, without the transfer of the aminobutyl moiety to any substrate, thereby producing *Δ* ^1^‐pyrroline (Fig. 3B). Such a Spd cleavage has been described for human DHS to occur [42]. In this case, Dap increase would be misleading. To distinguish between a Dap increase due to Spd cleavage alone or eIF5A modification, we compared the enzyme ability to utilize Spd in the presence and absence of the eIF5A precursor (Fig. 5). Additionally, we provide a method to detect deoxyhypusine after acid hydrolyses of the *in vitro* assay as a proof of concept. This method, however, requires several additional steps and is more time‐consuming.

### Effects of the assay buffer

Although we could establish a reproducible derivation procedure of polyamines in both assay buffers, the buffer indirectly affected the *in vitro* assays as it had an impact on detection sensitivity. Derivatization blank measurements proved to be important, as previously mentioned by Jámbor and Molnár‐Perl [35] and revealed that FMOC–acetone impurities interfere with the detection of Put and Spd, thereby impairing the sensitivity of the detection of these metabolites. Even more importantly, one impurity co‐elutes with Dah, a finding that disqualifies Dah as an internal standard. These impurities are especially relevant if glycine‐based assay buffer is used, and thus, higher amounts of FMOC have to be applied to achieve full and reproducible derivatization of the polyamines. Thus, the borate‐based assay buffer should be favored. However, the activity of an enzyme can considerably differ when tested in two distinct buffer systems, even if they share the same pH and concentration [43]. Various reasons might be responsible for this behavior [43]. Thus, the most suitable buffer has to be determined for the studied enzyme. We have shown that, for characterizing the DHS enzyme and its paralog, both tested buffer systems are suitable. The specific activity of *Sv*DHS and *Sv*HSS differ somewhat in the two different assay buffers, but the main features such as substrate preferences and promiscuity are the same, independent of buffer systems used.

### New insights into *Sv*DHS and *Sv*HSS activity

The new assays were used to characterize *Sv*DHS and *Sv*HSS, two enzymes that were previously studied in detail in a glycine‐based assay buffer and in assays that measured the incorporation of radioactivity from ^14^C‐labeled substrates [14]. With the new assays, we were able to confirm the previous results. HSS and DHS could be unequivocally distinguished because of their characteristic substrate preference. The specific activities for the aminobutylation of the eIF5A precursor of *Sv*DHS were, in the past, calculated to be 58 pkat·mg^−1^ [14]. In the new assay, *Sv*DHS showed an approx. 10‐fold higher activity with 338 and 440 pkat·mg^−1^, in the borate and glycine buffers, respectively. On the other hand, the specific activity for Hspd production of the *Sv*HSS was previously reported to be 784 pkat·mg^−1^ [14]. With 970 and 289 pkat·mg^−1^ in the borate and glycine buffers, respectively, the specific activity was in the same order of magnitude in the new assay. These observed differences can be attributed to the completely different methodologies. In the former DHS assay, eIF5A‐bound radioactivity after protein precipitation was used to calculate specific activity. Two important factors in that assay were the complete precipitation of the eIF5A and the thorough removal of remaining radiolabeled precursor. In the former HSS assay, radio thin‐layer chromatography was used, and radioscans were performed with a linear radiation counter tube detector for quantification.

However, an unexpected and previously undescribed observation was that *Sv*DHS uses, in the absence of the eiF5A precursor, Put and Spd as aminobutyl acceptors, thereby producing Hspd and Can in parallel. In the former assays, only Hspd was detected; this might have been because of the poor resolution of Hspd and Can in the previously used thin‐layer chromatography. Additionally, with the new assay, *Sv*HSS was shown to perform, in both, the presence and the absence of the eIF5A precursor, the transfer of aminobutyl from Spd to Spd, thereby producing Can, although at a slow rate. However, less Can was formed compared with Dap, indicating a cleavage of Spd in parallel and the release of Dap and Δ^1^‐pyrroline. This pattern is not affected by the omission or addition of the eIF5A precursor and is thus fully in agreement with the previous finding that the eIF5A precursor does not interact with the *Sv*HSS at all [14].

The finding that *Sv*HSS mainly cleaves Spd when Put is not available, whereas *Sv*DHS uses both Put and Spd as aminobutyl acceptors in the HSS assay, clearly indicates, on the one hand, that *Sv*DHS is more promiscuous concerning the aminobutyl acceptor than *Sv*HSS and, on the other hand, that *Sv*HSS is clearly optimized exclusively to produce Hspd. Nevertheless, if *Sv*DHS is incubated with equal amount of aminobutyl acceptors (eIF5A precursor, Put, Spd), the aminobutylation of the eIF5A precursor is favored. The mechanistic background of this preference is unclear. The aminobutyl acceptors compete for the same binding site in the active tetramer. To date, it has been postulated that, although four active sites are formed in the interface of the tetrameric DHS, only a single eIF5A precursor can be bound per tetramer [12, 44, 45]. A regulatory conformational change upon binding the cofactor NAD and one or both of the substrates has previously been suggested [9, 12].

## Conclusion

The new assay represents a powerful tool for characterizing DHS by directly quantifying the polyamine products after derivatization. This approach can be transferred to study further enzymes involved in polyamine metabolisms. In the biochemical characterization of DHS and its paralogs, the new assay allows the detection of side reactions that might occur in parallel to the main reaction. As DHS is a key regulator in human diseases, and as the targeting of DHS for drug development can be considered a promising therapeutic strategy, the new assay can be used to study the detailed action of potential inhibitors of DHS activity. Furthermore, in most eukaryotes, DHS is encoded by a single‐copy gene. However, in plants, repeated independent duplications of the DHS followed by functional divergence gave rise to HSS. Because of the increasing numbers of plant genomes that have been sequenced, additional DHS paralogs have been identified. For example, two DHS paralogs have been identified in *Lolium perenne* [46]. The biochemical characteristics of most of the newly identified paralogs are unknown. As DHS is involved in the central pathways of life, the characterization of these paralogs, including their side activities, will be of wide general interest.

## Materials and methods

### Chemicals

Solvents used for HPLC‐DAD‐FL (acetonitrile, tetrahydrofuran, acetone) were HPLC gradient grade, polyamines (1,3‐diaminopropane hydrochloride, putrescine dihydrochloride, 1,7‐diaminoheptane dihydrochloride, spermidine trihydrochloride, spermine tetrahydrochloride), and FMOC chloride, *N,N*‐dimethylformamide, and piperidine hydrochloride were obtained from Sigma‐Aldrich. Homospermidine was kindly provided by Thomas Stegemann (Biochemical Ecology and Molecular Evolution Group, Botanical Institute and Kiel Botanic Gardens, Christian‐Albrechts‐University, Kiel, Germany). 0.2 m stock solutions were prepared in deionized water and stored at −20 °C. Working solutions were prepared fresh from the individual stock solutions. Chemicals used for LC‐MS (water, acetonitrile, sodium formate) were of MS grade and purchased from VWR International GmbH (Darmstadt, Germany).

### Bioinformatic tools to calculate structure‐based predictions of polyamines and proteins

The ‘Chemicalize’ online platform (https://chemicalize.com/) and the protparam web tool on ExPASy [47] were used to calculate structure‐based predictions of polyamines (pKa, isoelectric point) and proteins (specific extinction coefficient, isoelectric point), respectively.

### Statistical analyses

Statistics for Fig. 5 and plotting the results was done using r (v3.6.1) [48] and the plyr (v1.8.6) [49] and ggplot2 (v3.3.2) [50] packages.

### Heterologous expression in *E. coli* and purification of DHS, HSS, and eIF5A precursor protein from *S. vernalis*

The open reading frames of the genes encoding DHS, HSS, and eIF5A precursor protein from *S. vernalis* were cloned into the pET22b vector (Novagen), which encodes an artificial C‐terminal hexahistidine (6xHis) tag extension, as described in Refs [21, 32]. The resulting constructs were heterologously expressed in *Escherichia coli* BL21(DE3) according to Ober and Hartmann [21, 32]. Recombinant proteins were purified using nickel‐nitrilotriacetic acid metal affinity chromatography (Qiagen, Hilden, Germany) according to the manufacturer’s protocol. For biochemical characterization, the purified proteins were concentrated and rebuffered in (a) glycine buffer (50 mm glycine solution adjusted to pH 9 with NaOH) or (b) borate buffer (50 mm boric acid solution adjusted to pH 9 with NaOH). Both buffers were supplemented with DTT (1 mm) to protect enzymatically relevant cysteine residues and EDTA (0.1 mm) as the complexing agent. The protein concentration was calculated from the sample’s absorption at 280 nm and the specific extinction coefficient of the respective protein (*Sv*DHS ɛ = 42 900, *Sv*HSS ɛ = 41 410, eIF5A precursor ɛ = 3230). Protein purity was monitored by SDS/PAGE analysis, for staining PageBlue Protein Staining Solution (Thermo Fisher Scientific, Waltham, MA, USA) was used.

### Activity assays of recombinant DHS and HSS stained

Two enzyme assays were routinely used. In the so‐called ‘DHS assay’, the enzyme’s ability to use the recombinant eIF5A precursor from *S. vernalis* as an aminobutyl acceptor was analyzed. Spd was used as the aminobutyl donor. The formation of Dap (Fig. 1) was quantified as one of the products of the enzyme’s activity. In the so‐called ‘HSS assay’, the enzyme’s ability to form Hspd was tested by using Spd and Put as aminobutyl donor and acceptor, respectively (Fig. 3). The reaction mixtures of the DHS assay are based on those described by Ober and Hartmann [32] and contained 10–80 µg DHS or HSS enzyme, 2 mm NAD^+^, 40 µm Spd, and 40 µm eIF5A precursor in the borate or glycine buffer. The reaction volume was 140 µL. The reaction mixtures of the HSS assay were identical to the DHS assay, except that both substrates, namely Spd and Put, were used at a concentration of 400 µm according to Ober and Hartmann [21]. The reaction mixtures were incubated at 30 °C for up to 32 min. To ensure linearity of reaction product formation over time, the enzyme amount and incubation time were adjusted, and 30 µL aliquots were taken after 1, 2, 4, 8, 16, and 32 min of incubation. To stop the reaction and to precipitate the protein, each aliquot was immediately mixed with 60 µL acetonitrile [51] containing diaminoheptane (Dah) and/or Cad as internal standards (ISTD) in equimolar concentrations to the polyamine substrates in the assay, that is, 13 and 133 µm, respectively, after precipitation. To improve protein precipitation, samples were placed on ice for 20 min. Precipitated protein was removed by centrifugation for 10 min at 21 000 ***g*** at room temperature. The supernatant was used for derivatization prior to HPLC‐UV/FL analyses.

### Derivatization, HPLC system, and conditions

The supernatant of the precipitated assay aliquots containing the soluble reaction products, that is, the polyamines, was derivatized with FMOC. The derivatization reaction was optimized according to Jámbor and Molnár [35]. For samples from assays in borate buffer, a 10 mm FMOC stock solution in acetonitrile was prepared and stored at 4 °C. The working solutions of 0.5 mm FMOC for DHS assay samples and 2.5 mm FMOC for HSS assay samples were diluted in acetone *prior* to use, because of the higher reaction rate of the amine groups with FMOC in acetone as a solvent [35]. Samples from assays in glycine buffer were derivatized with 25 mm FMOC in acetone, which was prepared freshly. Derivatizations following optimized conditions were as follows: 30 µL supernatant was mixed with 36 µL borate buffer (0.6 m, pH 8) and 72 µL FMOC in the acetone working solution, thereby providing excess FMOC (Table 3). The molar ratio of the reactants was [FMOC]:[substrate] = 30 : 1 for samples from DHS assays in borate buffer, 15 : 1 for HSS assay in borate buffer, and 4 : 1 for DHS and HSS assays in glycine buffer. The incubation time of the samples in borate buffer was 5 min at room temperature. For samples in glycine buffer, the derivatization time was extended to 10 min. Subsequently, piperidine was added to react with any excess FMOC (Table 3). Finally, the derivatization was stopped by adding a solution consisting of acetonitrile/sodium acetate buffer (400 mm, pH 5)/tetrahydrofuran (80/20/10; v/v/v). Tetrahydrofuran can be replaced by *N,N*‐dimethylformamide. 10–20 µL of this derivatization mix was then analyzed by HPLC‐FL (DHS assays) or HPLC‐UV (HSS assays). Polyamines were quantified via their relative peak area to the ISTD. The relative area was computed as percentage of the area of the ISTD. The calibration curves were generated by plotting the relative peak areas against the respective concentrations. Of note, the intensity of the UV absorption and fluorescence emission of the polyamines is expected to depend on the amount of attached FMOC moieties in the derivatized molecule. Therefore, the tetraamine Spm was used to provide a calibration curve for estimating the amount of the tetraamine Can.

**Table 3:** Optimum derivatization conditions for samples from DHS and HSS assays in borate‐ or glycine‐based assay buffers. Polyamine concentration was 40 µm in the DHS assay and 400 µm in the HSS assay. Samples from assays in glycine buffer also included 50 mm glycine as a reactant of FMOC.

	Borate buffer (0.6 m, pH 8) (µL)	Sample (i.e., assay aliquot) (µL)	FMOC	Piperidine	Stop (µL)
DHS assay (borate assay buffer)	36	30	72 µL (0.5 mm)	1.8 µL (20 mm)	60
HSS assay (borate assay buffer)	36	30	72 µL (2.5 mm)	4.5 µL (40 mm)	60
DHS and HSS assays (glycine assay buffer)	36	30	72 µL (25 mm)	9 µL (200 mm)	60

An Ultimate 3000 system with a LPG‐3400SD pump, a WPS‐3000 autosampler, a DAD‐3000 diode array detector, and a FLD‐3100 fluorescence detector (Thermo Fisher Scientific, Waltham, MA, USA) was used. The UV absorbance of derivatized polyamines was measured at 270 nm. For fluorescence detection, the excitation wavelength was fixed at 265 nm, and the emission was monitored at 310 nm as described by Huhn *et al*. [36]. Chromeleon version 7.2 (Thermo Fisher Scientific) was used to operate the HPLC system and to integrate the chromatograms. An Accucore C18‐XL reversed‐phase column (Thermo Fisher Scientific, dimensions: 250 × 3 mm, particle size 4 µm) was employed with a mobile phase consisting of 40 mm sodium acetate buffer at pH 5 (eluent A) and acetonitrile (eluent B). The following gradient profile was used: 50% B for 15 min, to 60% B in 10 min, to 69% B in 9 min, to 71% B in 12 min, to 78% B in 7 min, and to 95% B in 7 min, which was held for another 7 min. The flow rate was 0.6 mL·min^−1^.

### LC‐MS

LC‐MS analysis was performed on a Shimadzu Nexera X2 system with an LC‐30AD binary pump, connected to a SIL‐30AC autosampler, CTO‐20AC column heater, SPD‐M30A diode array detector, and a Shimadzu LC‐MS 8030 triple quadrupole mass spectrometer. MS spectra were recorded in electron spray ionization (ESI) mode, scanned in a range of *m*/*z* = 100–1300 in the positive and negative ionization modes. Separation was accomplished by using the above‐mentioned HPLC column and 40 mm sodium formate buffer (pH 5, solvent A) and acetonitrile with the following gradient: 50% B for 15 min, to 69% B in 19 min, to 72% B in 12 min, to 82% in 7 min, and to 95% B in 10 min, which was held for another 10 min. The postrun time was set to 10 min and the column temperature to 25 °C. The flow rate was 0.6 mL·min^−1^, and spectra were recorded from 10 to 73 min.

### Acid hydrolyses and analyses of deoxyhypusine

*Sv*DHS was incubated with the eIF5a precursor protein and Spd under typical DHS assay conditions. As negative control, *Sv*DHS and eIF5A precursor proteins were incubated, but without Spd. After 32 min of incubation at 30 °C, 60 µL of the assays was hydrolyzed with 120 µL 6 m HCl overnight at 110 °C. Basic amino acids (arginine, histidine, lysine, deoxyhypusine) and polyamines were extracted from the hydrolyzed assay reaction via Strata™‐X‐C 33 µm Polymeric Strong Cation exchanger columns (Phenomenex, Torrance, CA, USA) following the manufacturer’s protocol except an additional wash step with 0.1 m borate buffer, pH 8.8. The basic amino acids were eluted with 5% NH_4_OH in methanol. For derivatization with FMOC, the solvent was evaporated overnight and the sample was resolved in 30 µL borate‐based assay buffer (50 mm boric acid solution adjusted to pH 9 with NaOH). After mixing the sample with 60 µL acetonitrile including 20 µm Cad as internal standard, 30 µL of the mixture was derivatized according to the HSS assay in borate assay buffer protocol (Table 3) to provide excess FMOC to allow full derivatization of the basic amino acids plus the polyamines in the reaction mixture. The derivatized samples were analyzed with HPLC‐FLD as described above with the following modified gradient: 16% B to 38% B in 15 min, to 50% B in 10 min, to 60% B in 4 min, and to 95% B in 26 min, which was held for another 10 min. For LC‐MS analysis (instrumentation as described above), 40 mm ammonium acetate buffer (pH 5, solvent A) and acetonitrile (solvent B) were used at a flow rate of 0.3 mL·min^−1^ with the following gradient: 16% B to 38% B in 30 min, to 50% B in 20 min, to 60% in 8 min, and to 95% B in 52 min, which was held for another 20 min. The postrun time was set to 20 min and the column temperature to 30 °C. Spectra were recorded from 5 to 10 min. MS spectra were recorded as described above. Arginine, histidine, and lysine were identified via commercially available reference samples. Deoxyhypusine was identified via its mass spectrum.

## Conflict of interest

The authors declare no conflict of interest.

## Author contributions

DO proposed and discussed the project. EK, ASP, and SSC designed and conducted experiments, and interpreted the data. EK prepared the figures, and designed and wrote the manuscript. DO wrote, discussed, and edited the manuscript. ASP and SSC discussed and edited the manuscript.

## Supporting information


**Fig. S1.** Box‐plots displaying the peak area of polyamine standards (Dap, Put, Spd, Hspd) relative to the ITSD (Cad) after independent FMOC derivatization reactions. 1A. Polyamines derivatized under HSS assay conditions (400 µM each, UV detection), 1–4 in borate buffer, 5–6 in glycine buffer. 1B. Polyamines derivatized under DHS assay conditions (40 µM each, FLD detection), 1–4 in borate buffer, 5–6 in glycine buffer.

**Fig. S2.** Expression and purification of recombinant HSS, DHS, and eIF5A from *S. vernalis*. Shown is a PageBlue stained SDS‐polyacrylamide gel of the purified and concentrated *Sv*HSS (42 kDa, *lane* 2), *Sv*DHS (42 kDa, *lane* 3), and *Sve*IF5A (18 kDa, *lane 4*). *Lane 1*. PageRuler Prestained Protein Ladder. *Lane 5*. PageRuler Unstained Protein Ladder.

**Fig. S3.** Full mass spectrum of Canavalmine.

## Data Availability

The data supporting the conclusions of this article are included within the article (and its additional files).
