# Supplementary figures and images for: Development of an activity assay for characterizing deoxyhypusine synthase and its diverse reaction products
Source: FEBS Open Bio. 2020 Dec 8;11(1):10–25. doi: 10.1002/2211-5463.13046 (PMC7780104; doi:10.1002/2211-5463.13046)

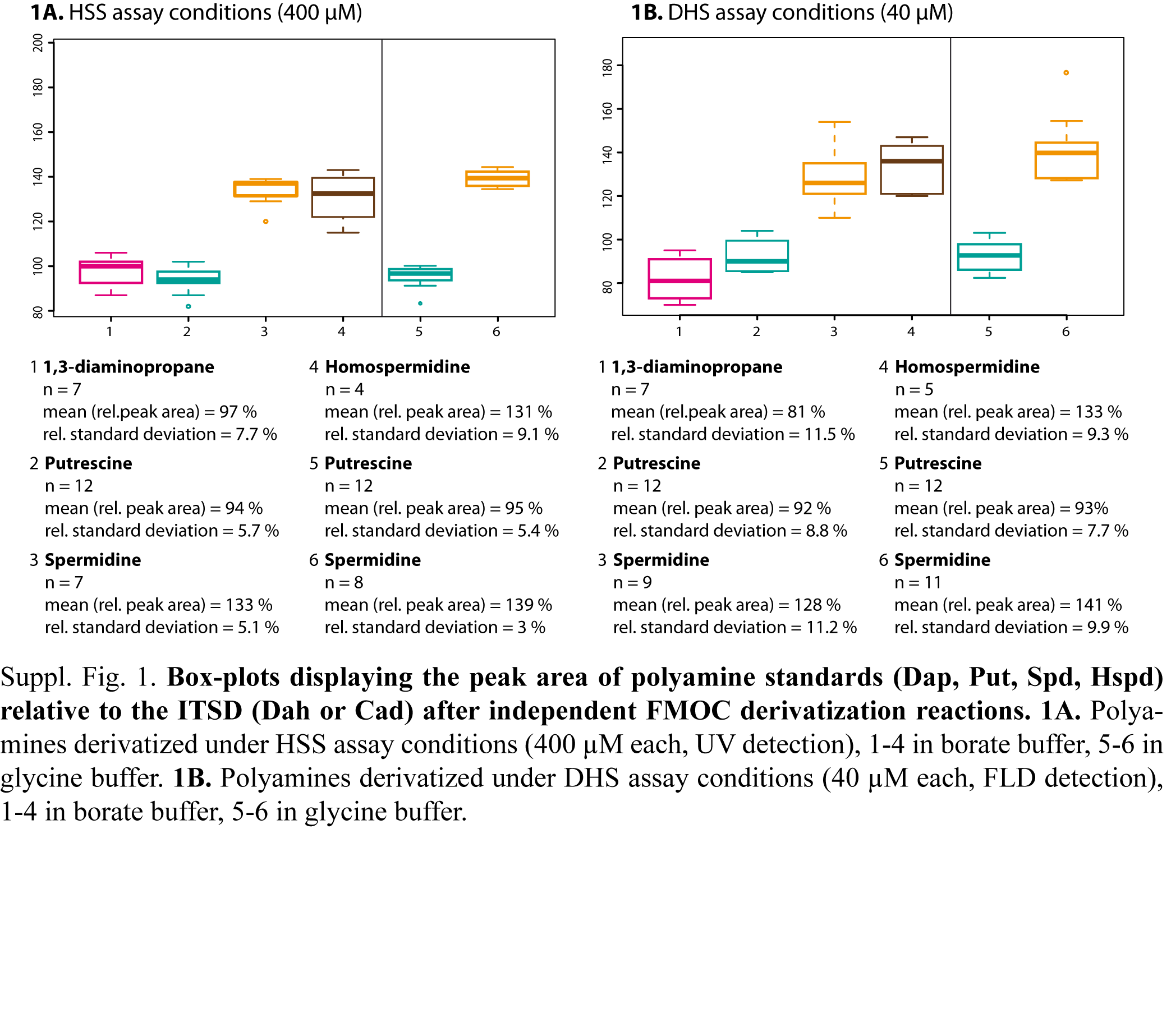

Supplement: Supplementary file 1 — Fig. S1. Box‐plots displaying the peak area of polyamine standards (Dap, Put, Spd, Hspd) relative to the ITSD (Cad) after independent FMOC derivatization reactions. 1A. Polyamines derivatized under HSS assay conditions (400 µM each, UV detection), 1–4 in borate buffer, 5–6 in glycine buffer. 1B. Polyamines derivatized under DHS assay conditions (40 µM each, FLD detection), 1–4 in borate buffer, 5–6 in glycine buffer. [file FEB4-11-10-s001.tif]

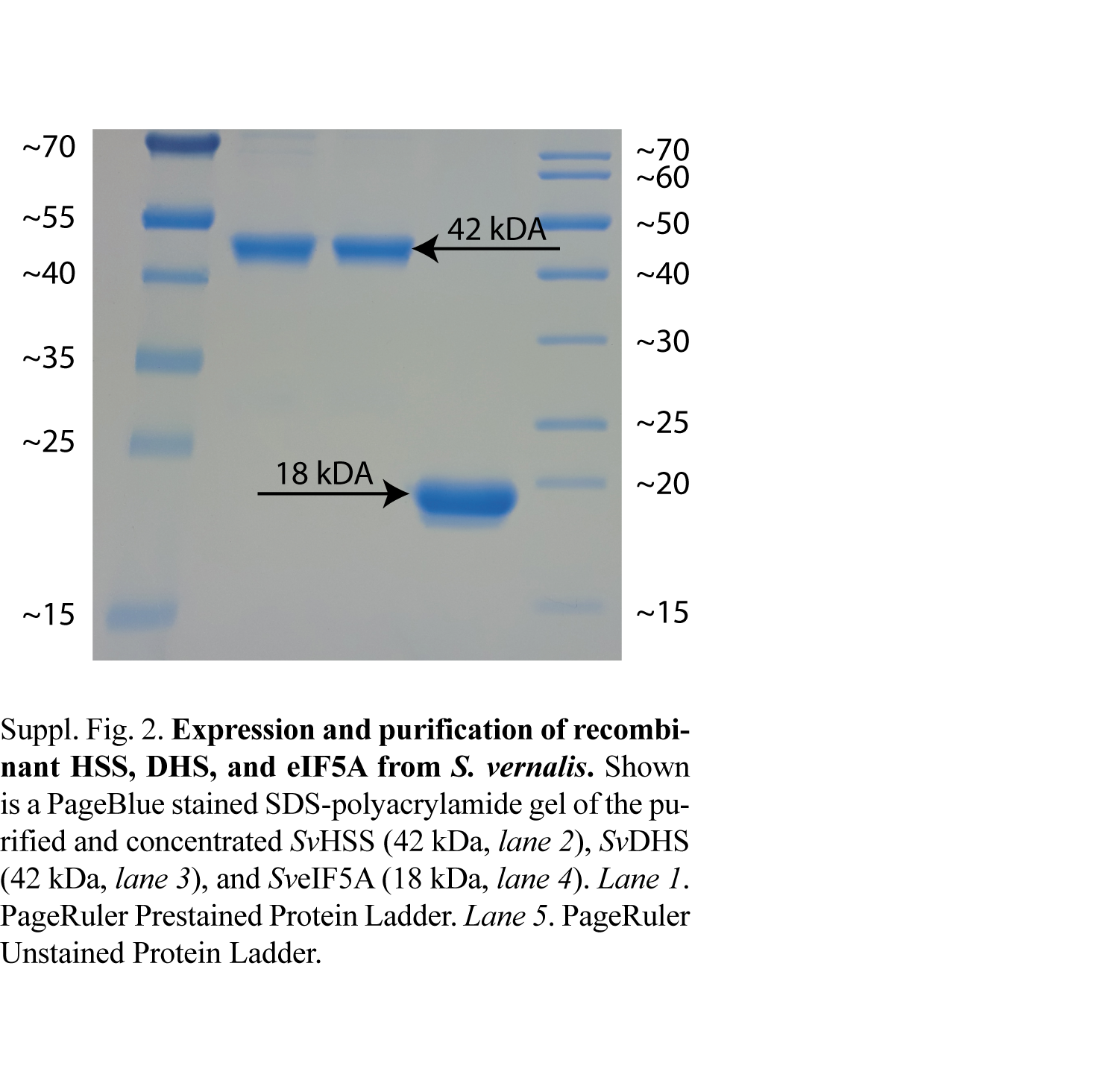

Supplement: Supplementary file 2 — Fig. S2. Expression and purification of recombinant HSS, DHS, and eIF5A from S. vernalis. Shown is a PageBlue stained SDS‐polyacrylamide gel of the purified and concentrated SvHSS (42 kDa, lane 2), SvDHS (42 kDa, lane 3), and SveIF5A (18 kDa, lane 4). Lane 1. PageRuler Prestained Protein Ladder. Lane 5. PageRuler Unstained Protein Ladder. [file FEB4-11-10-s002.tif]
