# Supplementary material for: Development of an activity assay for characterizing deoxyhypusine synthase and its diverse reaction products
Source: FEBS Open Bio. 2020 Dec 8;11(1):10–25. doi: 10.1002/2211-5463.13046 (PMC7780104; doi:10.1002/2211-5463.13046)

Suppl. Fig. 3. **Full mass spectrum of canavalmine**

==== Shimadzu LabSolutions Browser Report ====

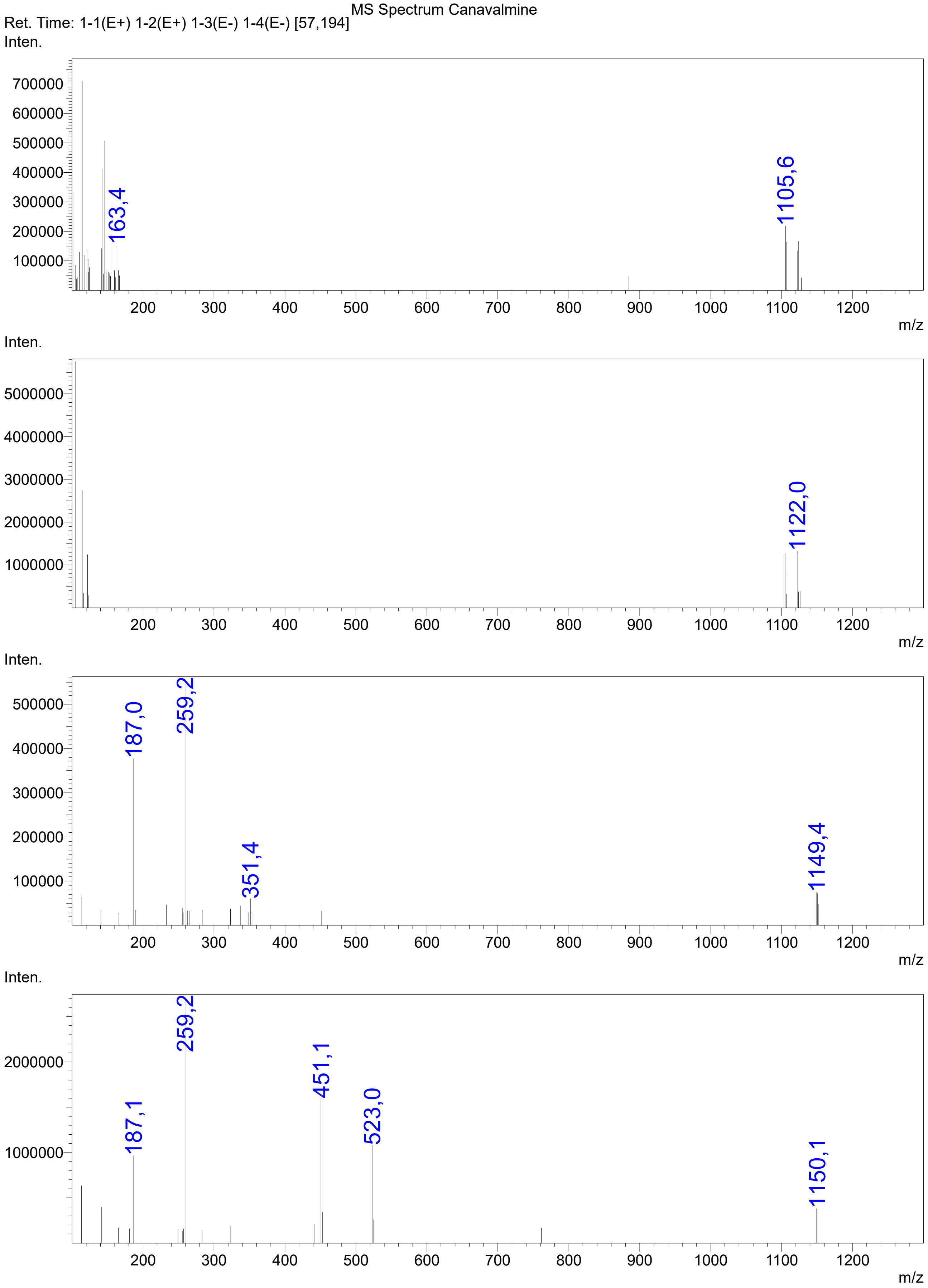

Supplement: Supplementary file 3 — Fig. S3. Full mass spectrum of Canavalmine. [file FEB4-11-10-s003.pdf]
